# Supplementary material for: Enhanced Antibacterial Activity through Silver Nanoparticles Deposited onto Carboxylated Graphene Oxide Surface
Source: Nanomaterials (Basel). 2022 Jun 7;12(12):1949. doi: 10.3390/nano12121949 (PMC9228250; doi:10.3390/nano12121949)
Supplement: Supplementary file 1 [file nanomaterials-12-01949-s001.zip › nanomaterials-1735674-supplementary.pdf]

nanomaterials

MDPI

Supplementary Materials

Enhanced Antibacterial Activity through Silver Nanoparticles Deposited onto Carboxylated Graphene Oxide Surface

Arturo Barjola [1], María Ángeles Tormo-Mas [2,*], Oscar Sahuquillo [1], Patricia Bernabé-Quispe [2], José Manuel Pérez [2] and Enrique Giménez [1,*]

[1] Instituto de Tecnología de Materiales, Universitat Politècnica de València (UPV), Camino de Vera s/n, 46022 Valencia, Spain; arbarrui@doctor.upv.es (A.B.); ossana@upvnet.upv.es (O.S.)

[2] Severe Infection Group, Health Research Institute La Fe, University and Polytechnic La Fe Hospital, Avda. Fernando Abril Martorell 106, 46026 Valencia, Spain; patricia_bernabe@iislafe.es (P.B.); manuel_perez@iislafe.es (J.M.P.)

* Correspondence: tormo_man@iislafe.es (M.Á.T.-M.); enrique.gimenez@mcm.upv.es (E.G.)

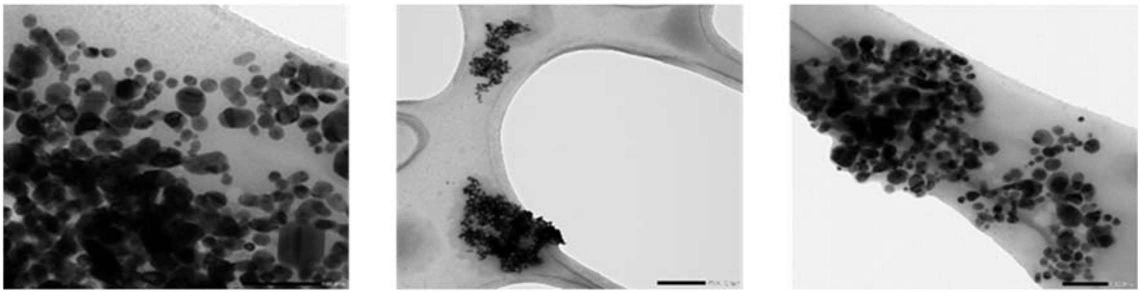

Figure S1. TEM micrographs of AgNps.

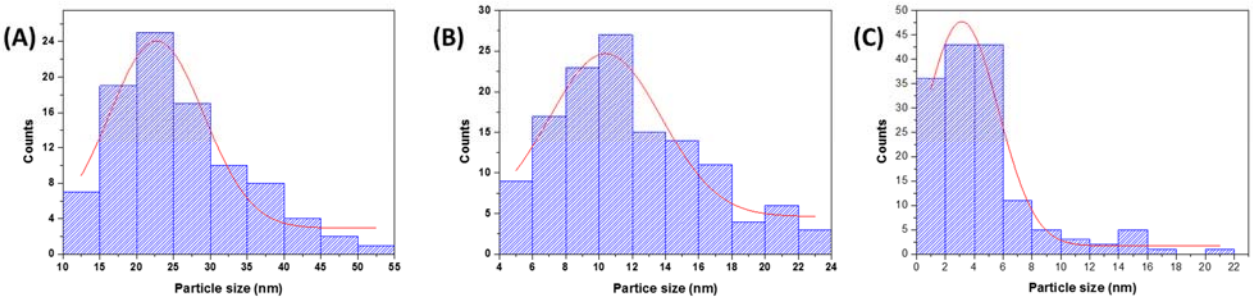

Figure S2. Histograms and Gaussian fit for the particle size of (**A**) AgNPs, (**B**) GO-Ag, and (**C**) GOCOOH-Ag silver nanoparticles.

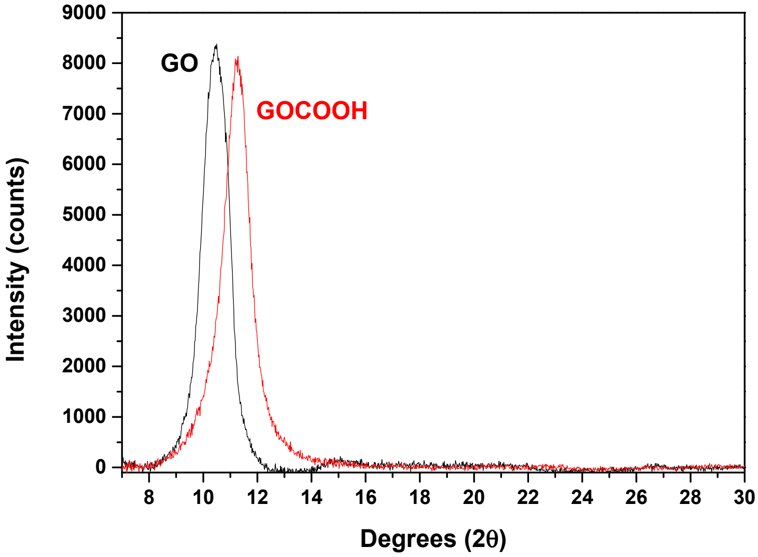

Figure S3. XRD spectrum for GO (black) and GOCOOH (red).

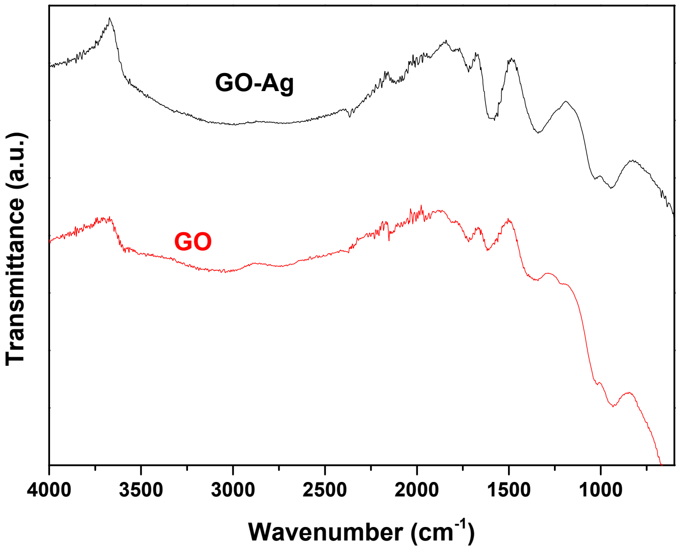

Figure S4. FTIR spectrum GO (red) and GO-Ag (black).

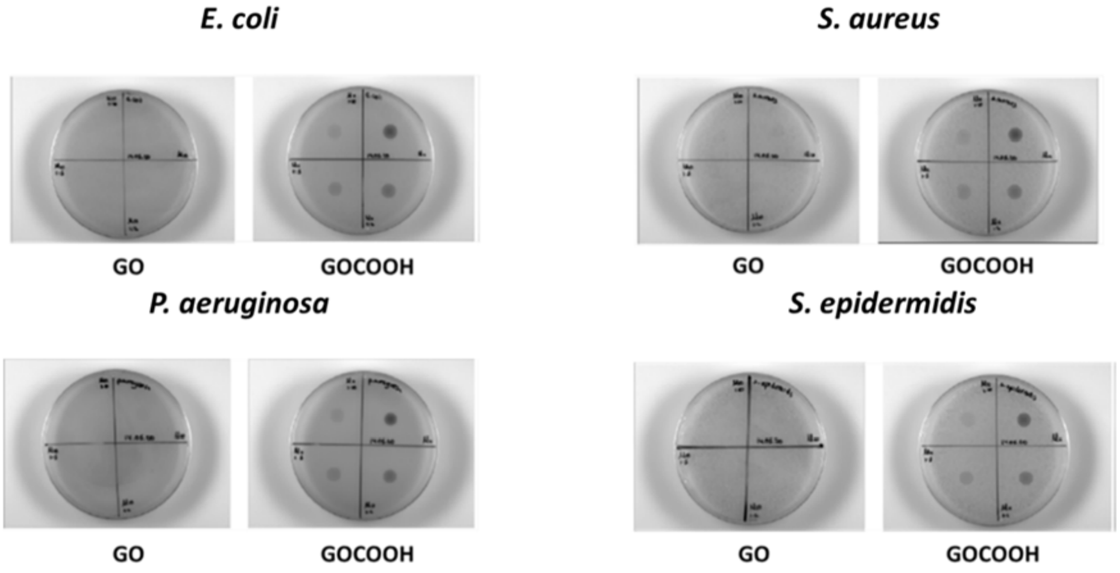

Figure S5. Kirby–Bauer plate diffusion method applied for GO and GOCOOH initial screening.

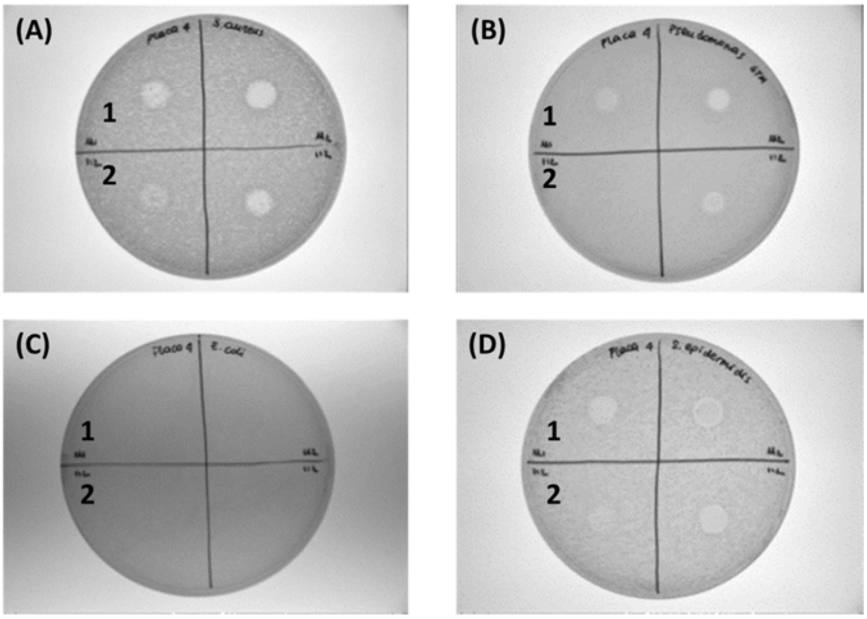

Figure S6. Kirby–Bauer plate diffusion method applied for AgNP initial screening. (**A**) *S. aureus,* (**B**) *P. aeruginosa,* (**C**) *E.coli*, and (**D**) *S. epidermidis.* AgNP concentration was 25 µg/mL for (A1, B1, C1, D1) and 12.5 µg/mL for (A2, B2, C2, D2).

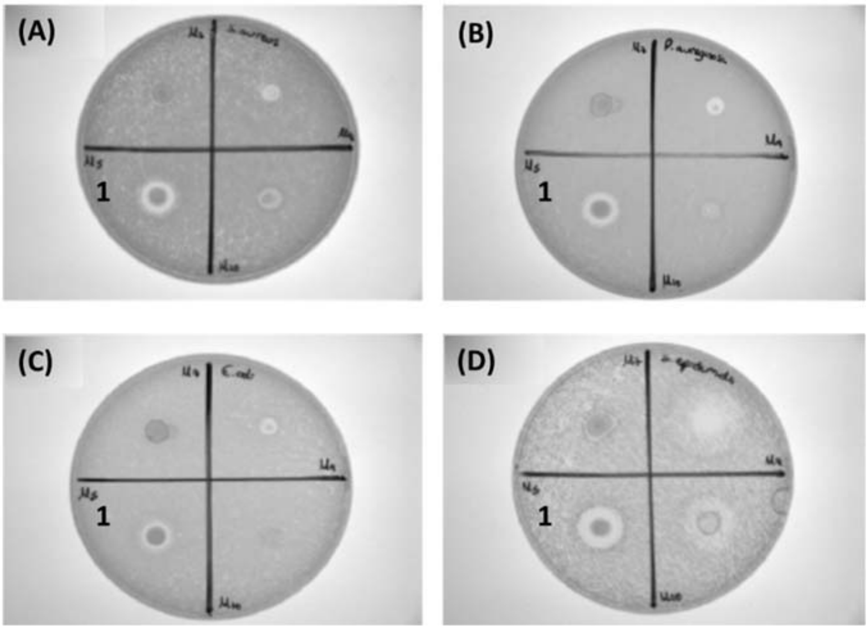

Figure S7. Kirby–Bauer plate diffusion method applied for GO-Ag initial screening. (**A1**) *S. aureus,* (**B1**) *P. aeruginosa,* (**C1**) *E.coli,* and (**D1**) *S. epidermidis.* AgNP concentration was 12.5 μg/mL.

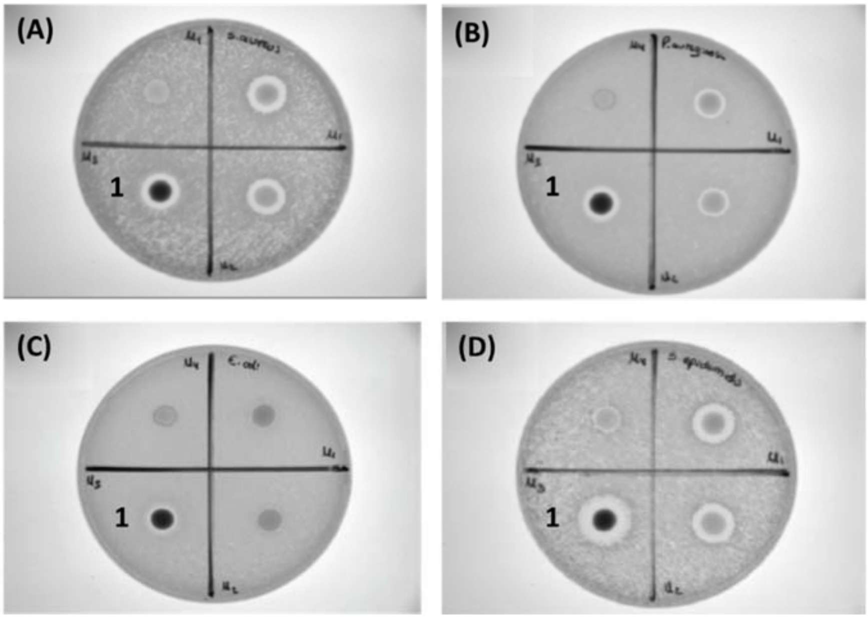

Figure S8. Kirby–Bauer plate diffusion method applied for GOCOOH-Ag initial screening. (**A1**) *S.aureus,* (**B1**) *P. aeruginosa,* (**C1**) *E.coli,* and (**D1**) *S. epidermidis.* AgNP concentration was 12.5 μg/mL.

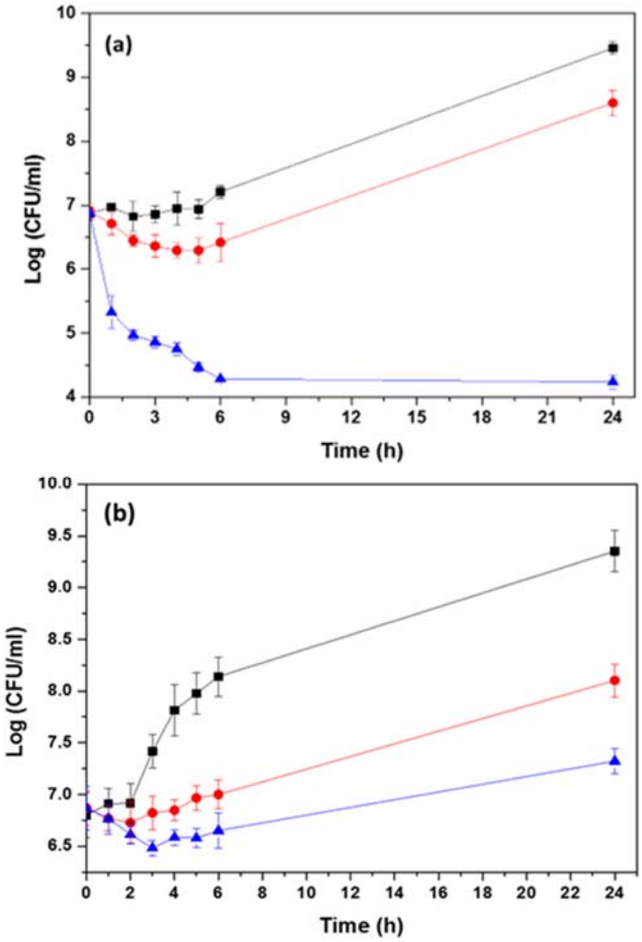

Figure S9. Growth curves of (**a**) *S. aureus* ATCC25423 and (**b**) *S. epidermidis* ATCC32984 in the presence of GO-Ag (red circles) and GOCOOH-Ag (blue triangles) at 12.66 μg/mL. Control is depicted with black squares. The data were presented as means and SD of at least three independent experiments.

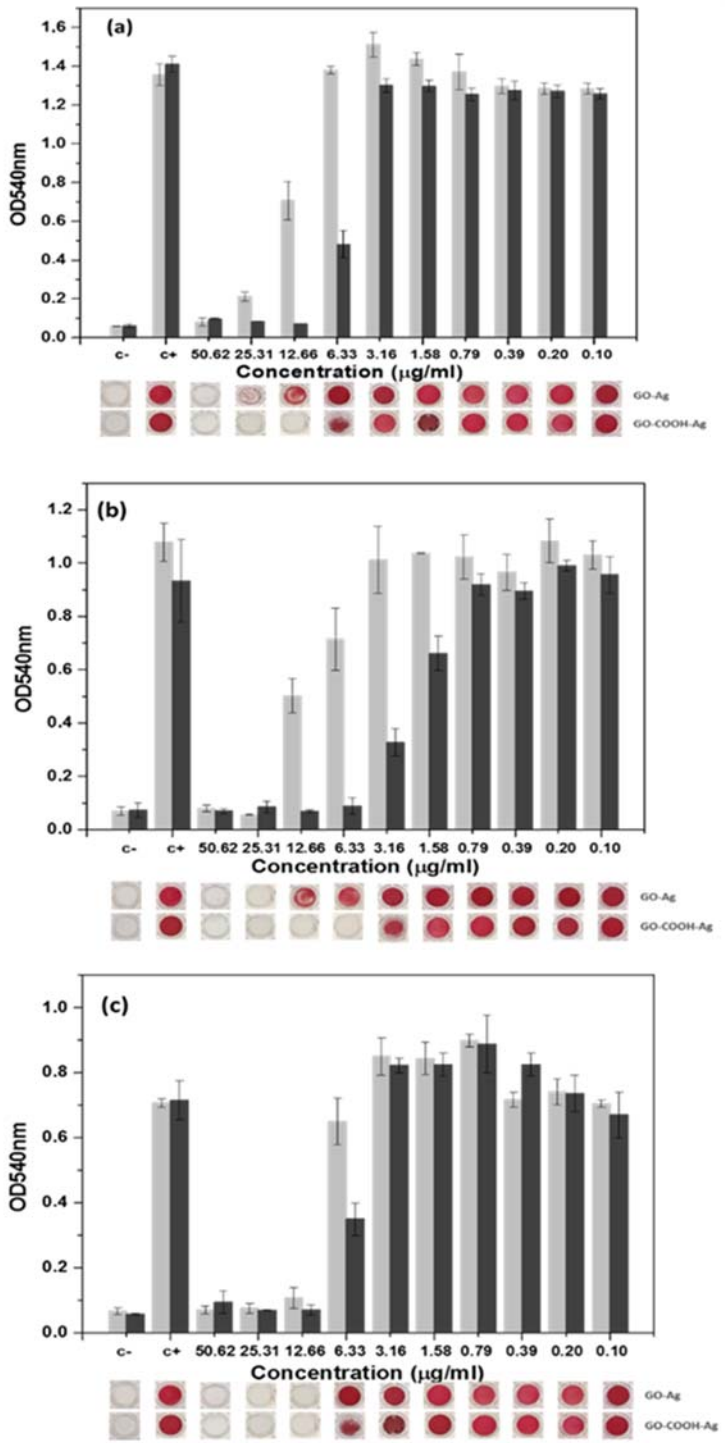

Figure S10. Effect of GO-Ag (light grey) and GOCOOH-Ag (dark grey) hybrid nanomaterials on the biofilm of (**a**) *S. epidermidis* RP62A; (**b**) *P. aeruginosa* PFQ2, and (**c**) *E. coli* ATCC25922. Quantification (upper panel) and images (lower panel) of the biomass of a 48 h biofilm adhering in microtiter plates; optical density after staining with 0.1% safranin was measured at 540 nm.

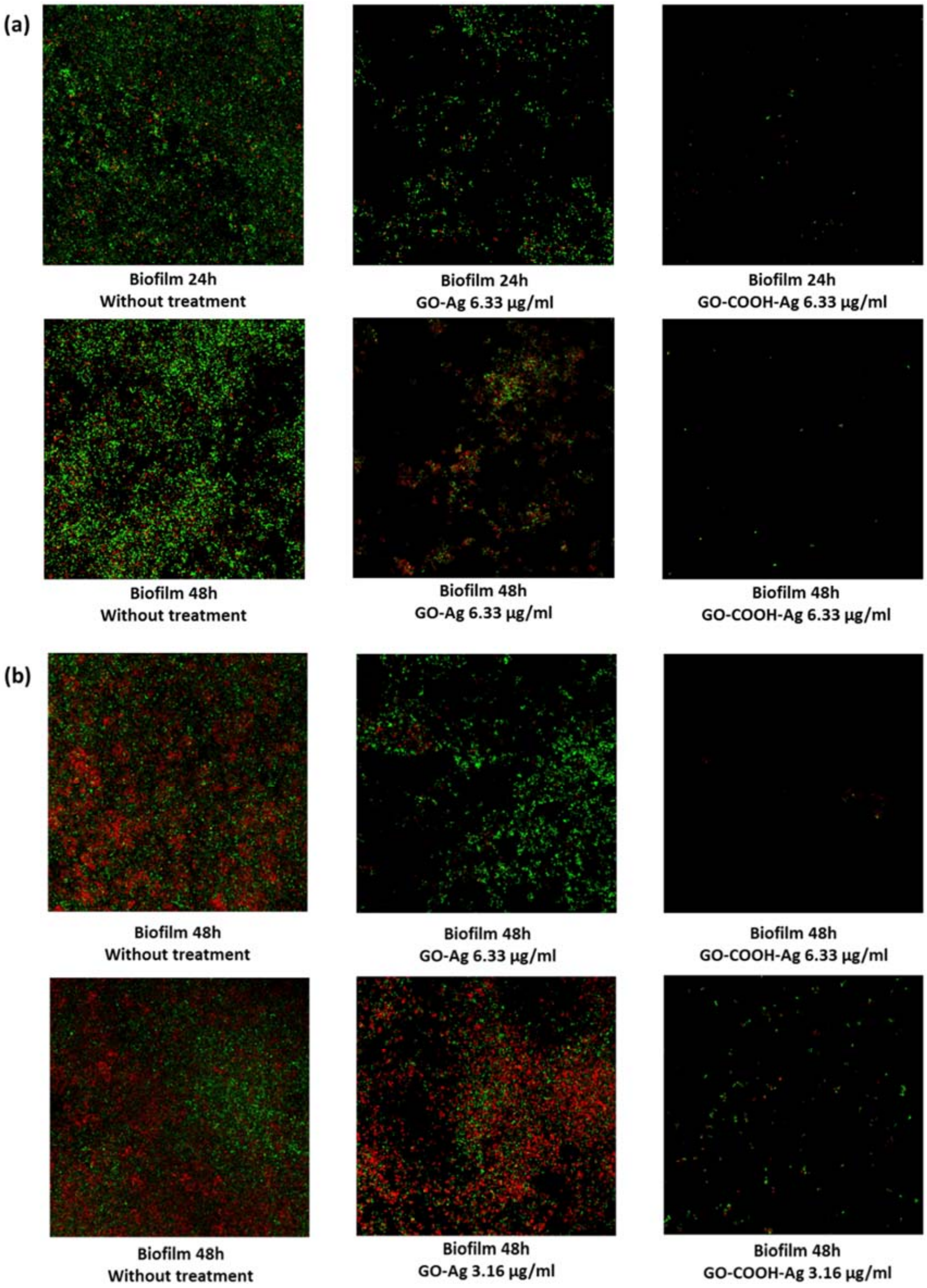
(a)
Biofilm 24h
Without treatment
Biofilm 24h
GO-Ag 6.33 µg/ml
Biofilm 24h
GO-COOH-Ag 6.33 µg/ml
Biofilm 48h
Without treatment
Biofilm 48h
GO-Ag 6.33 µg/ml
Biofilm 48h
GO-COOH-Ag 6.33 µg/ml
(b)
Biofilm 48h
Without treatment
Biofilm 48h
GO-Ag 6.33 µg/ml
Biofilm 48h
GO-COOH-Ag 6.33 µg/ml
Biofilm 48h
Without treatment
Biofilm 48h
GO-Ag 3.16 µg/ml
Biofilm 48h
GO-COOH-Ag 3.16 µg/ml

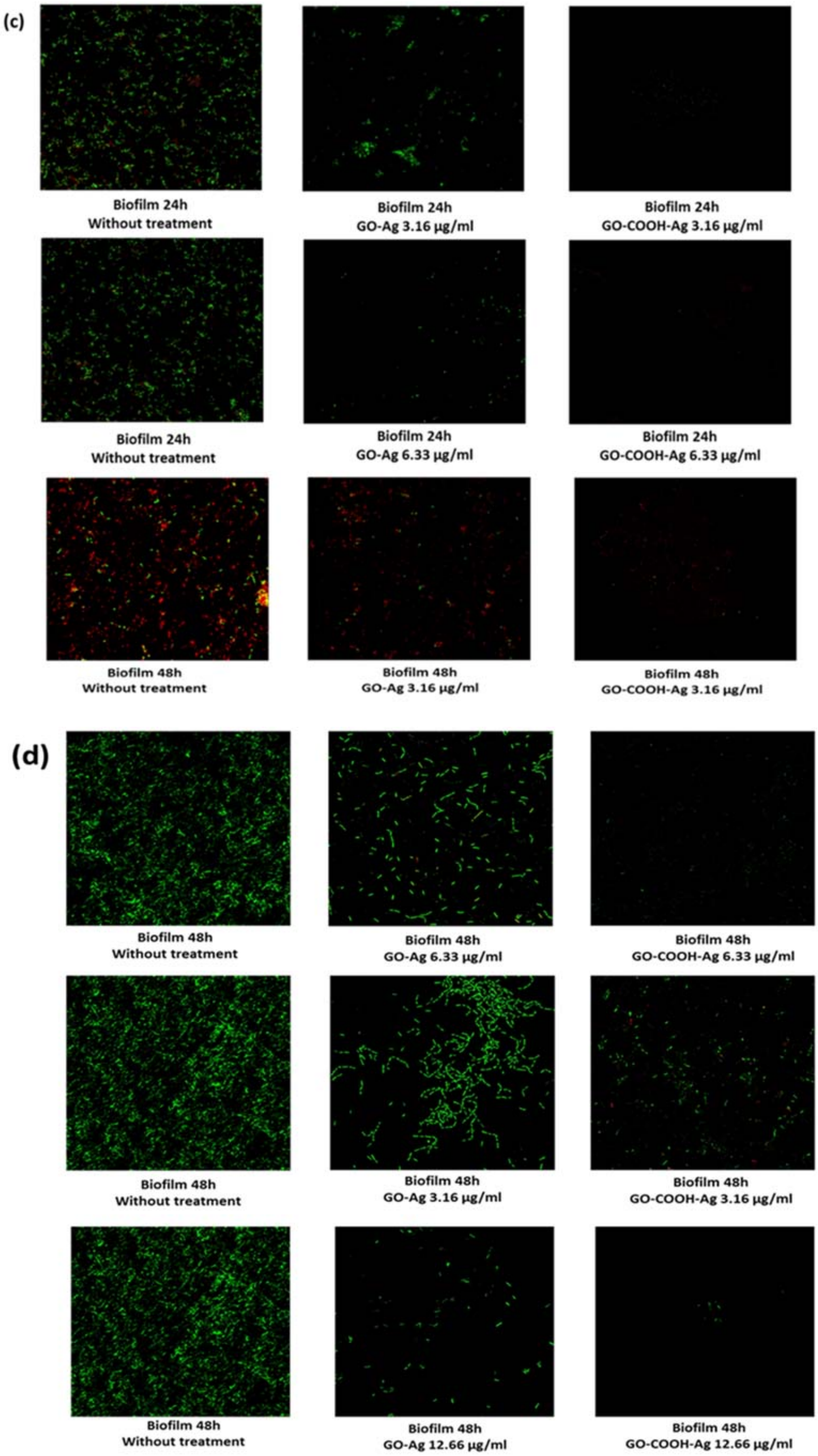

Figure S11. Confocal microscopy images biofilm (40× oil objective) of (**a**) *S. aureus* V329, (**b**) *S. epidermidis* RP62A, (**c**) *P. aeruginosa* PFQ2, and (**d**) *E. coli* ATCC25922.
